# Arraying of microphotosynthetic power cells for enhanced power output

**DOI:** 10.1038/s41378-022-00361-7

**Published:** 2022-03-14

**Authors:** Kiran Kuruvinashetti, Muthukumaran Packirisamy

**Affiliations:** Optical-Bio Microsystems Laboratory, Department of Mechanical, Industrial and Aerospace Engineering, Concordia University, Montreal, QC H3G1M8 Canada

**Keywords:** Electrical and electronic engineering, Engineering, Nanoscale devices

## Abstract

Microphotosynthetic power cells (µPSCs) generate power through the exploitation of living photosynthetic microorganisms by harvesting sunlight. The thermodynamic limitations of this process restrict the power output of a single µPSC. Herein, we demonstrate µPSCs in four different array configurations to enhance power output from these power cells. To this effect, six µPSCs were arrayed in series, parallel, and combinations of series and parallel configurations. Each µPSC was injected with a 2 mL liquid culture of photosynthetic microorganisms (Chlamydomonas reinhardtii) in the anode and 2 mL of 25% (w/v) electron acceptor potassium ferricyanide (K_3_Fe(CN)_6_) in the cathode. The combinations of µPSCs connected in series and parallel generated higher power than the individual series and parallel configurations. The combinations of six µPSCs connected in series and in parallel produced a high power density of 1914 mWm^−2^ in the presence of white fluorescent light illumination at 20 µEm^−2^s^−1^. Furthermore, to realize the array strategy for real-time applications, a 1.7 V/2 mA rating light-emitting diode (LED) was powered by combinations of series and parallel array configurations. The results indicate the reliability of µPSCs to produce electricity from photosynthetic microorganisms for low-power applications. In addition, the results suggest that a combination of microlevel photosynthetic cells in array format represents a powerful optimal design strategy to enhance the power output from µPSCs.

## Introduction

Microphotosynthetic power cells (µPSCs), also known as biophotovoltaics, are emerging as promising renewable power sources. This technology exploits photosynthetic microorganisms to harvest energy from sunlight^1–4^. Thus, µPSCs facilitate addressing the present concern of sustainable energy generation. In µPSCs, light energy is converted to electrical power with high-energy charge-separated electron-hole pairs by exploiting living photosynthetic microorganisms such as cyanobacteria and blue–green algae^3^. The excited electrons are transferred across a series of complex intracellular electron carriers, and eventually, a fraction of these electrons is exported across the cell membrane and released into the external environment^3^. The µPSC, in turn, harnesses these electrons through the anode and cathode electrodes, thereby generating electricity^1,3,5–12^.

Although sunlight-to-chemical energy conversion efficiency is relatively low at 0.1%, the primary source, solar energy, is nearly infinitely available^6,13^. To this end, it is essential to understand the limiting factors that impede performance in several directions such as understanding microorganisms at the cellular level, electrochemical engineering design, and fabrication of µPSCs to harness more energy from sunlight.

One of the significant restraints and limitations of the µPSC is its low power density. Consequently, to address these issues, quite a few approaches have been studied. Algal biofilms^14^ and genetically engineered cyanobacteria^15^ were utilized for electricity generation^14^. Innovative engineering design strategies have been demonstrated for the enhancement of power density by decoupling storage and power delivery for µPSCs^3^. Subsequently, mediator-free microfluidic µPSCs with cyanobacterial cells have been utilized to generate a power density up to 100 mW/m^2^^16^. Digital printed cyanobacteria were also used to generate electricity^17^. Hence, in this context, several studies exist on various dimensioned single-cell µPSCs and methods to increase their performance^3,6,8,18–21^. Although microlevel µPSCs outperform macrolevel µPSCs due to their high surface area to volume ratio, higher columbic efficiency, lower internal resistance, higher mass transfer efficiency, and smaller distance or no distance between the electrodes^1,3,6,18^, they are thermodynamically limited^22^. Thermodynamically, the maximum open circuit voltage (V_oc_) that could be produced by µPSCs with photosynthetic microorganisms is only 1.8 V^22^. Consequently, arraying µPSCs is one of the possible optimal solutions to obtain the desired voltage and current output from the µPSC such that it can be utilized to power low- and ultralow-power devices.

In this context, a commercial inkjet printer was utilized to fabricate a thin-film paper-based biophotovoltaic cell consisting of a layer of cyanobacterial cells on top of a carbon nanotube conducting surface. A peak power density of 0.38 ± 0.07 mWm^−2^ and a current density of 4 mAm^−2^ were generated under 100 µEm^−2^s^−1^ light illumination. Subsequently, µPSC arraying strategies were realized to power real-time low-power devices. An arrayed configuration was also used to obtain an overall effective voltage of 1.4–1.5 V and current output of 1.5–2 µA to power a commercial digital clock^17^. Thereafter, to obtain the desired voltage of 3 V from the µPSC, another array configuration was utilized to power light-emitting diodes (LEDs).

Although a few works have investigated array configurations for real-time applications, detailed analyses are still under investigation. Such a detailed analysis of the array configurations provides insights into the design of suitable low-power converters for real-time low- and ultralow-power devices. Herein, we demonstrate an array of µPSCs in series, parallel, and a combination of series and parallel connections. The 12 µPSCs were fabricated and connected in various array configurations. Electrical parameters such as the open circuit voltage (V_oc_), short circuit current (I_sc_), load voltage (V_L_), and current (I_L_) at 1 kΩ and 0.5 kΩ were recorded for all configurations. Furthermore, the polarization curve (I-V) and power-current (I-P) characteristics were analyzed. To demonstrate the array strategy for real-time low-power applications, an LED with a 2 mA current rating and a 1.7 V voltage rating were powered from an array of µPSCs.

### Device design and operation

**Fig. 1:**
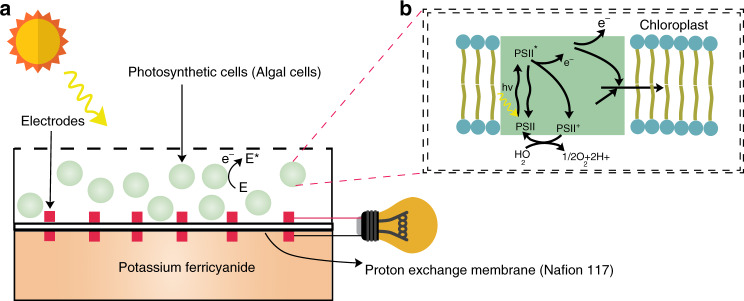
Schematic of the µPSC. **a** The anode and cathode chambers are separated by a proton exchange membrane. **b** Mechanistic schematic of photoexcited electron transfer in algal cells and exoelectrogenic activity of algal cells

The µPSC anode and cathode chambers were fabricated from polydimethylsiloxane (PDMS), and both chambers were separated by a Nafion 117 proton exchange membrane. Nafion 117 was specifically chosen considering its robustness and a relatively high proton diffusion capacity^19^. The anode and cathode electrodes were fabricated on both sides of the Nafion membrane (Methods section). Such fabrication reduces the internal resistance between the electrodes, thereby increasing the performance by decreasing losses. After fabricating these components, all components were bonded together with PDMS. To seal the cathode compartment, microscopic cover glasses were employed. Then, 2 mL of 25% K_3_[Fe(CN)_6_] was injected into the cathode chamber. Subsequently, the anode chamber was also injected with 2 mL of suspension-cultured green algal cells, and the anode chamber was purposefully not closed to allow for the diffusion of carbon dioxide and oxygen from the atmosphere (Fig. 1a). The photosynthetic process in the cyanobacterial cell (algal cells) is shown in Fig. 1b.

**Fig. 2:**
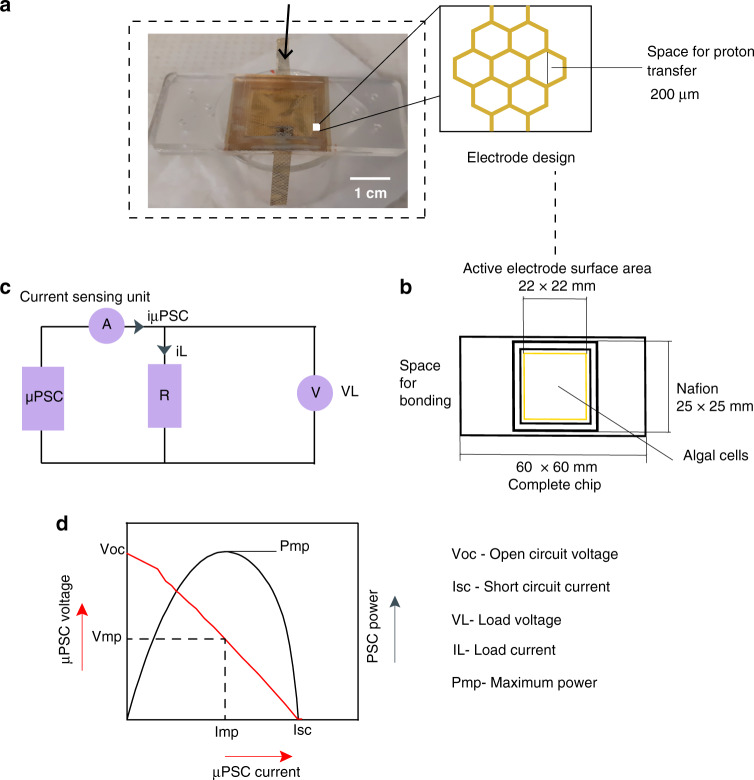
Design and performance of arrayed micro-photosynthetic power cells. **a** Photograph of the final assembled µPSC chip. The various components of µPSCs are shown in the supplementary information (Fig. S4). Schematic illustrating the electrode structures. Electrodes were sputtered with a 40 nm film of Au on both sides. A 200 µm space was provided for proton transfer through the proton exchange membrane. **b** The complete dimensions of the µPSC. The electrode surface area for the active reaction was 4.84 cm^2^. **c** Schematic illustrating the real-time electrical loading conditions and measurement of V_L_ and I_L_ using a current sensing unit specifically designed to measure the microlevel current and low voltages at high precision. **d** Typical polarization and power-current characteristics of the µPSC. V_oc_ represents the open circuit voltage, I_sc_ represents the short circuit current, and P_mp_ represents the maximum power. V_mp_ indicates the µPSC voltage at maximum power; I_mp_ indicates the µPSC current at maximum power

C. reinhardtii was used as a photosynthetic microorganism driven by its fast growth conditions and previously demonstrated exoelectrogenic activities^18,19^. Exoelectrogenic electrons follow various conduits to reach the electrode surface, such as by direct electron transfer and indirect electron transfer^1^. After injecting anolyte and catholyte, the current sensing unit that was specifically designed to read low currents was connected, and data were logged into the DAQ. For further polarization, power curves were recorded with 0–50 kΩ rheostat (Methods section). The typical polarization and power curves of the µPSC are shown in Fig. 2d.

Multistrand cables and mini copper alligator clips were used to connect the µPSC in an array configuration. For all the experimental investigations, a light illumination of 20 µEm^−2^s^−1^ was employed based on previous observations.

### Individual µPSC performance

Twelve individual µPSCs were fabricated. The fabrication of the single cell and its dimensions are illustrated in Fig. 2. Among these twelve µPSCs, numbers 1 to 6 were employed for the series and parallel configurations (Fig. S3(a and b)). The remaining µPSCs from 7 to 12 were employed for combinatory configurations CC-1 and CC-2.

The electrical performance of the µPSCs was highly dependent on the quality of the fabrication^18,19^. The individual V_oc_ and I_sc_ values of each µPSC were determined, as summarized in Table 1. (As the focus of the paper is the performance of array configurations, single-cell I-V and I-P characteristics and loading conditions are not presented.) However, for the performance of the individual µPSCs, readers can refer to our previous works^12,18,19,23–25^. Uniform light illumination of 20 µEm^−2^s^−1^ and an operating temperature of 23 °C were maintained as per our previous high-performance operating conditions for all µPSCs^17^. Table 1 demonstrates the electrical performance of all 12 individual µPSCs.

**Table 1:** Performance of individual µPSCs

	V_oc_ (mV)	Variation (mV)	I_sc_ (µA)	Variation (µA)
µPSC-1	775	12	400	6
µPSC-2	730	12	534	6
µPSC-3	810	15	1220	15
µPSC-4	720	12	800	10
µPSC-5	800	15	460	6
µPSC-6	770	12	410	6
µPSC-7	800	15	930	10
µPSC-8	770	12	820	10
µPSC-9	806	12	530	6
µPSC-10	810	15	600	6
µPSC-11	700	12	360	6
µPSC-12	800	15	815	10

### SA6 configuration

**Fig. 3:**
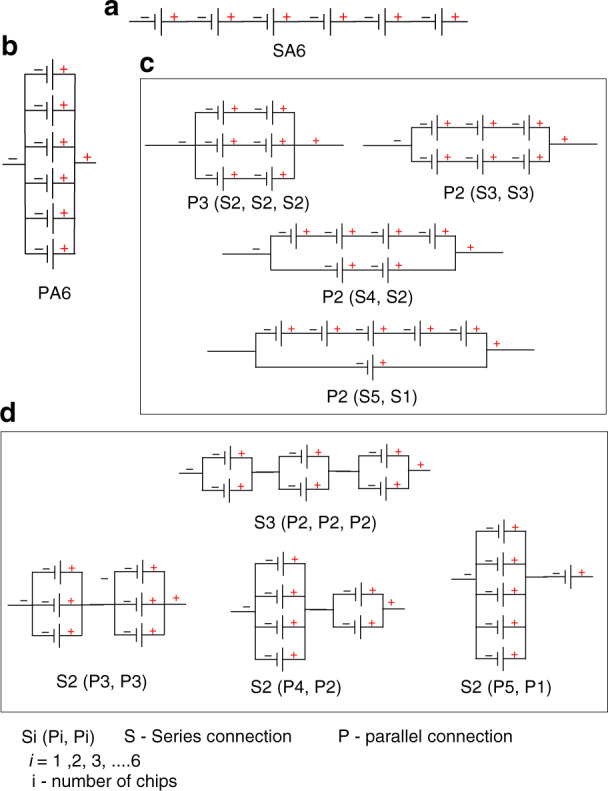
Arrayed configurations of micro-photosynthetic power cells. **a** SA6 configuration of µPSCs. **b** PA6 configuration of µPSCs. **c** Combinatory configuration-1 (CC-1). **d** Combinatory configuration-2 (CC-2)

In the SA6 configuration, the µPSCs were connected in series (Fig. 3a).

### PA6 configuration

In this configuration, the µPSCs were connected in parallel to observe the performance (Fig. S1).

### Complimentary configuration (CC-1)

To observe the performance of the µPSCs in combinations of series and parallel configurations, four unique configurations were chosen. The combinations used were [(S2, S2, S2)] (Fig. 3c), [P2 (S3, S3)], [P2 (S4, S2)] and [P2 (S5, S1)].

### Complimentary configuration (CC-2)

To observe the performance of the µPSCs in combinations of parallel and series configurations, four unique configurations were also chosen. The combinations used were [S3 (P2, P2, P2)] (Fig. 3d), [S2 (P3, P3)], [S2 (P4, P2)] and [S2 (P5, P1)].

During the connection of the different arrays, no sequence of µPSCs was followed mainly to observe the performance of the array configurations of randomly connected µPSCs. Figure S1a, b shows µPSCs utilized in the array configurations. For the CC-1 and CC-2 configurations, all six µPSCs (from 7 to 12, Table 1) were connected for all combinations. A photograph of the CC-1 [P2 (S3, S3)] configuration and CC-2 [S2 (P3, P3)] configuration is shown in Fig. S1e, f, respectively.

## Results and discussions

### Open circuit voltage (V_oc_)

**Fig. 4:**
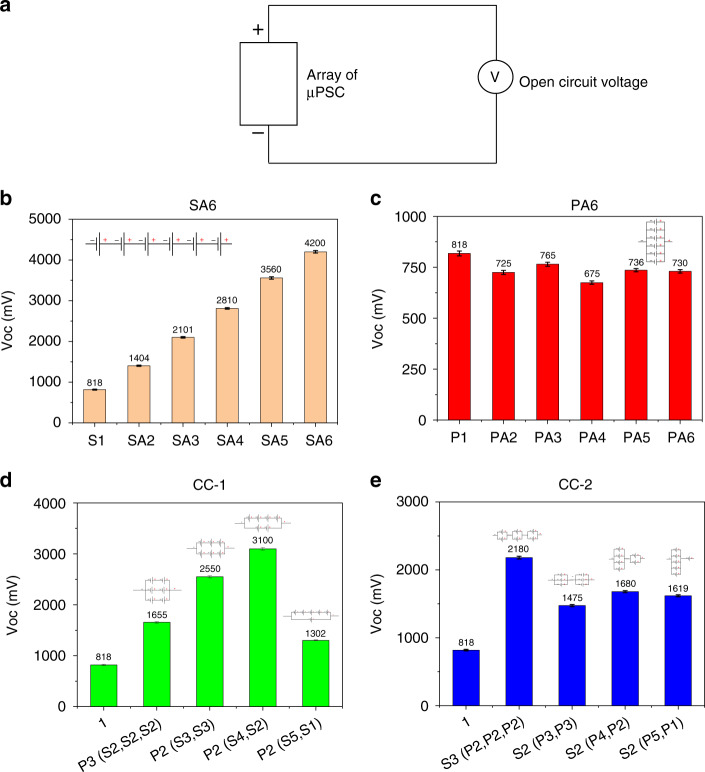
Open circuit voltage of arrayed micro-photosynthetic power cells. **a** Schematic demonstrating the measurement of V_oc_ for all configurations. **b** V_oc_ of the SA6 configuration. **c** V_oc_ of the PA6 configuration. **d** V_oc_ of the CC-1 configuration. **e** V_oc_ of the CC-2 configuration

Figure 4a depicts a schematic of the V_oc_ data logging of all array configurations. Figure 4b shows the V_oc_ of the SA6 configuration of µPSCs. After reaching a steady-state response, the V_oc_ was recorded for approximately 5 minutes for each combination. However, in our previous demonstrations, stable long-term performance for 20–24 hours was provided^19^. In the SA6 configuration, the effective V_oc_ was observed as the summation of individual µPSC V_oc_ values. A V_oc_ of 4200 mV was obtained for six µPSCs connected in series (Fig. 4b). Owing to the ohmic losses, their effective terminal voltage V_oc_ was not the linear summation of terminal voltages of individual µPSCs. The alligator clips, connecting cables, contributed to ohmic losses, which led to an increase in the total resistance of the µPSC connection. In contrast, in the PA6 configuration, the V_oc_ remained almost identical to that of individual µPSCs. Among the configurations, the overall effective V_oc_ was the same as that of the lowest µPSC V_oc_ in that connection. Although the V_oc_ value was essentially identical, it exhibited a very small variation range from 725 mV to 765 mV. Among the CC-1 configurations, the [P2 (S4, S2)] combination showed a higher V_oc_ of 3100 mV than the other 3 configurations. In contrast, the CC-2 configuration [S3 (P2, P2, P2)] showed a higher V_oc_ (2180 mV) than the other combinations. Therefore, among the four array configurations, the SA6 configuration generated a higher V_oc_.

### Short circuit current (I_sc_)

**Fig. 5:**
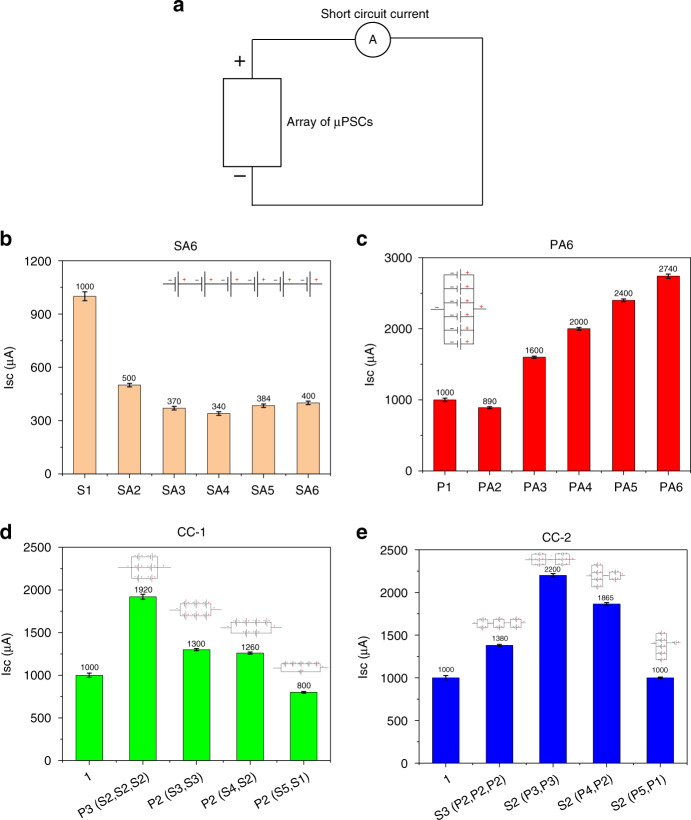
Short circuit current of arrayed micro-photosynthetic power cells. **a** Schematic demonstrating the measurement of I_sc_ for all configurations. **b** I_sc_ of the SA6 configuration. **c** I_sc_ of the PA6 configuration. **d** I_sc_ of the CC-1 configuration. **e** I_sc_ of the CC-2 configuration

Figure 5a illustrates a schematic of I_sc_ data logging from the array of µPSCs. In the SA6 configuration, the effective I_sc_ was that of the lowest performing (I_sc_) µPSC (Fig. 5b). For instance, in the SA1 configuration, the first µPSC showed an I_sc_ of 1000 µA. However, in the SA2 configuration, the effective I_sc_ dropped to 500 µA because of the poor performance of the 2nd µPSC. Similar observations were made with the SA3, SA4, SA5, and SA6 configurations (Fig. 5b). The results indicate that by adding more µPSCs connected in series, their effective I_sc_ will remain that of the lowest performing µPSC.

In contrast, in the PA6 configuration, the effective I_sc_ was found to be the summation of all the individual I_sc_ values (losses are not measured). The PA2 configuration, which was the parallel connection of µPSCs 2 and 6 (from Table 1), demonstrated a lower I_sc_ of 890 µA. As both µPSC I_sc_ values were 534 and 410, their effective I_sc_ was the summation of both I_sc_ values. Similar performance was observed when more µPSCs were added in a parallel configuration. The I_sc_ increased with increasing number of µPSCs in a parallel configuration. For 6 µPSCs connected in parallel, an I_sc_ of 2740 µA was obtained. The results indicated that adding more µPSCs in a parallel configuration increased the effective I_sc_.

From the PA6 results, it was known that I_sc_ increased, representing an almost linear summation of the individual I_sc_ values; the lowest I_sc_ was found in SA6. The CC-1 and CC-2 configurations showed mixed results, as they had both series and parallel connections of µPSCs. In the CC-1 configuration, the [P3 (S2, S2, S2)] combination demonstrated a higher I_sc_ (1920 µA) than all other combinations (Fig. 5d). In the CC-2 configuration, [S2 (P5, P1)] showed a higher I_sc_ (2200 µA) than the other combinations (Fig. 5e).

### Electrical loading

To observe the performance of the µPSCs under real-time loading conditions, load tests at 1 kΩ and 0.5 kΩ were performed for all array configurations (Fig. S1c–f).

### Load voltage (V_L_) at 1 kΩ

**Fig. 6:**
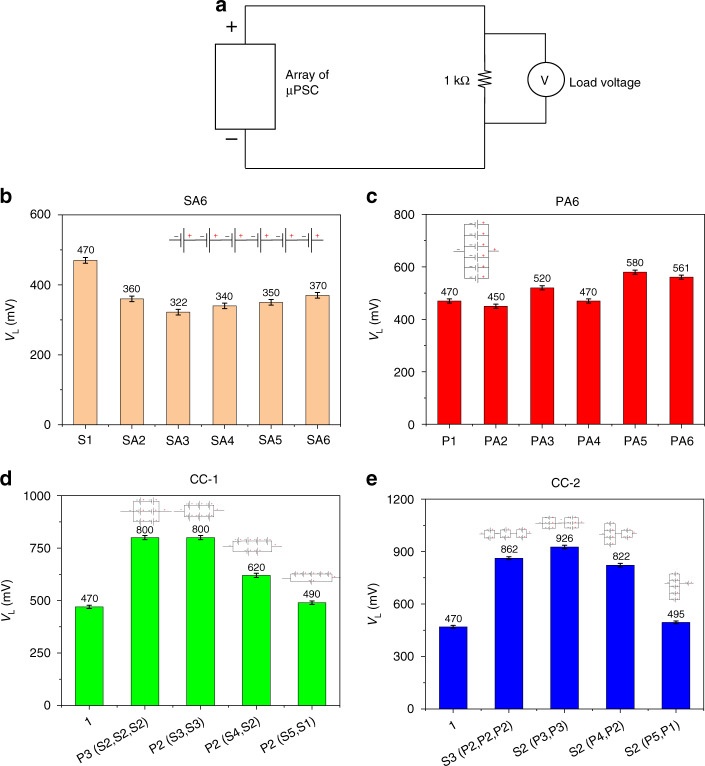
Load voltage of arrayed micro-photosynthetic power cells at 1 kΩ. **a** Schematic demonstrating the measurement of V_L_ for all configurations at 1 kΩ. **b** V_L_ of the SA6 configuration. **c** V_L_ of the PA6 configuration. **d** V_L_ of the CC-1 configuration. **e** V_L_ of the CC-2 configuration

Figure 6a depicts a schematic of data logging of the loading conditions for the array configurations. In the SA6 configuration, all array combinations from 2 µPSCs in series to 6 µPSCs in series demonstrated a lower V_L_ than the single µPSC V_L_ (470 mV), perhaps due to the lower effective I_sc_ of the SA6 configuration. As the effective I_sc_ of all SA6 configurations is much lower than that of the single µPSC (high performing), their effective is V_L_ also lower. For the SA6 configuration, the V_L_ value at 1 kΩ varied from 320 to 370 mV. The variation was highly dependent on the V_oc_ and I_sc_ of the µPSCs in the array configurations. The results indicate that in the SA6 configurations, having more µPSCs does not increase the V_L_. The reason is that the effective µPSC current flowing through the load resistance of 1 kΩ is very low (Fig. 6b). Thus, the V_L_ will not increase in this configuration.

For the PA6 configuration, the V_L_ was slightly higher than that of a single µPSC. For the parallel connection of 5 µPSCs, the V_L_ was slightly higher than that of other µPSCs connected in parallel. The reason could be the selection of µPSCs for this configuration. However, the V_L_ increase was not significant compared to that of a single µPSC. Again, here, the lower V_L_ was due to the lower effective V_oc_ of the PA6 configuration. Although the effective I_sc_ was higher in this configuration, due to the lower V_oc_ value, their effective V_L_ was slightly lower value than that of a single µPSC.

Overall, in the PA6 configuration, the V_L_ varied from 450 to 580 mV. Compared with the values obtained for the SA6 configuration, those of the PA6 configuration were slightly higher because of the marginally higher V_oc_ and I_sc_ of this configuration. Nevertheless, in both cases, the V_L_ of the SA6 and PA6 configurations was nearly identical to or slightly lower than that of a single µPSC.

In contrast to these SA6 and PA6 configurations, the CC-1 and CC-2 configurations showed higher V_L_ values. In the CC-1 configuration, the combinations [P3 (S2, S2, S2)] and [P2 (S3, S3)] demonstrated a higher V_L_ of 800 mV, which is 42.5% higher than the single µPSC V_L_ of 460 mV. It was indicated that whenever the V_oc_ and I_sc_ of the configurations were high, their effective V_L_ increased. However, for the combinations [P2 (S4, S2)] and [P2 (S5, S1)], a slightly lower V_L_ was observed, which is because of the lower effective I_sc_ of these configurations. In the CC-2 configuration, the combination [S2 (P3, P3)] generated a higher V_L_ than the other combinations. In this configuration, all the combinations showed a higher V_L_ than that of a single µPSC.

Among all four array configurations, CC-1 and CC-2 showed higher V_L_ values than the SA6 and PA6 configurations due to their higher effective V_oc_ and I_sc_. This result indicates that for real-time loading conditions, combinations of series and parallel connections are the ideal configurations.

### Load current (I_L_) at 1 kΩ

**Fig. 7:**
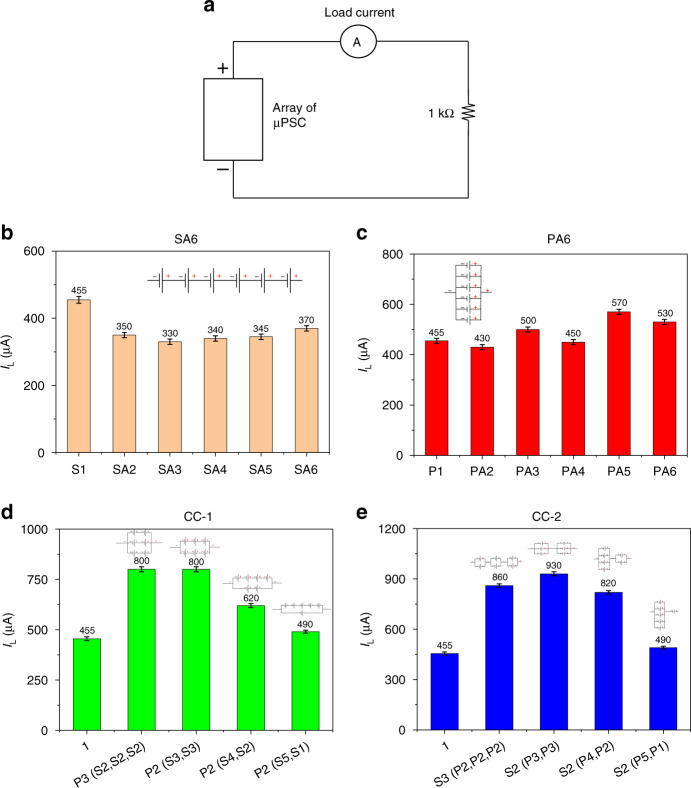
Load current of arrayed micro-photosynthetic power cells at 1 kΩ. **a** Schematic demonstrating the measurement of I_L_ for all configurations at 1 kΩ. **b** I_L_ of the SA6 configuration. **c** I_L_ of the PA6 configuration. **d** I_L_ of the CC-1 configuration. **e** I_L_ of the CC-2 configuration

Figure 7a shows a schematic of the load current at 1 kΩ for the different array configurations. In the SA6 configuration, the I_L_ of µPSCs from 2 µPSCs to 6 µPSCs in series was lower than the single µPSC I_L_ of 450 µA. In this configuration, the I_L_ at 1 kΩ varied from 330 to 370 µA. However, the variation in I_L_ with increasing number of µPSCs connected in series was insignificant. This finding indicates that having more µPSCs in series does not increase the I_L._ Again, the reason is the lower I_sc_ of the effective array configurations. Although the effective V_oc_ of the SA6 configuration was high due to its smaller effective I_sc_, the I_L_ was relatively low (Fig. 7b). In the PA6 configurations, the I_L_ was found to be slightly higher than that of a single µPSC. Five µPSCs connected in parallel demonstrated a marginally higher I_L_ value than other parallel connected µPSCs. However, the increase was insignificant compared to that of a single µPSC. The lower effective V_oc_ resulted in a lowered I_L_.

In the CC-1 configurations, the combinations [P3 (S2, S2, S2)] and [P2 (S3, S3)] demonstrated a higher I_L_ of 800 µA, whereas a single µPSC showed an I_L_ of 450 µA. In contrast, the combinations [P2 (S4, S2)] and [P2 (S5, S1)] showed a slightly lower I_L_ than the combinations [P3 (S2, S2, S2)] and [P2 (S3, S3)] because of the lower effective V_oc_ and I_sc_ of the array configurations. However, these values were higher than that of a single µPSC. In the CC-2 configurations, the combination [S2 (P3, P3)] generated a higher I_L_ (930 µA) than the other combinations. In addition, all the combinations showed higher I_L_ values than the single µPSC I_L_.

Among the array combinations, the CC-1 and CC-2 combinations showed a higher I_L_ than the SA6 and PA6 configurations. Therefore, for real-time loading applications, combinations are much more suitable than only series and parallel connections of µPSCs.

### Load voltage (V_L_) at 0.5 kΩ

To characterize the performance of the array configurations, another condition of a resistive load of 0.5 kΩ was chosen. In the SA6 configurations, from 2 µPSCs to 6 µPSCs, the array configuration demonstrated a lower V_L_ than the single µPSC V_L_ at 0.5 kΩ. Here, the V_L_ varied from 169 to 200 mV, slightly less than that of a single µPSC (300 mV). In the PA6 configuration, a slightly higher V_L_ was observed in comparison with that of a single µPSC. In this configuration, 5 µPSCs in parallel demonstrated a higher V_L_ than the other parallel configurations. However, the increase was insignificant compared to the single µPSC V_L_.

**Fig. 8:**
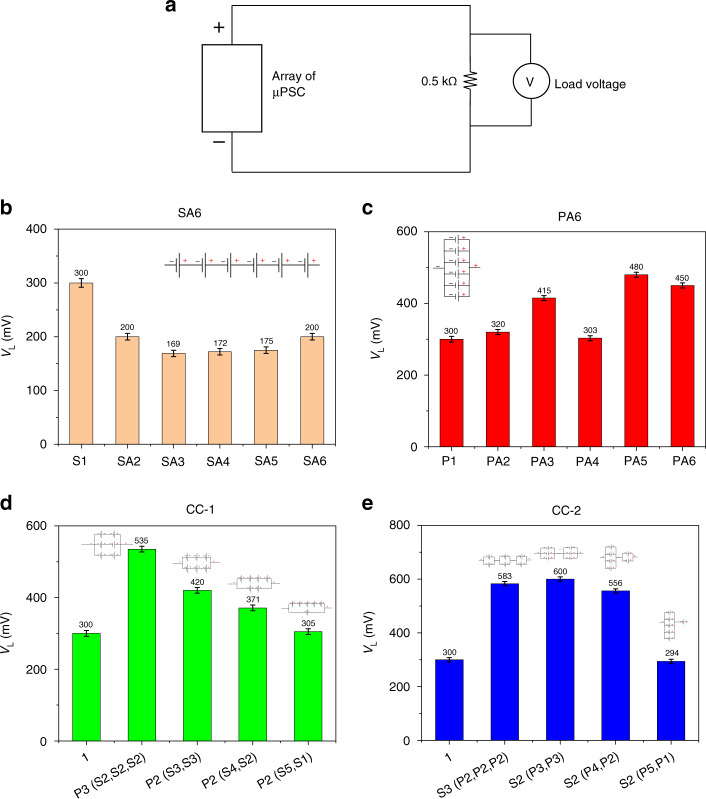
Load voltage of arrayed micro-photosynthetic power cells at 0.5 kΩ. **a** Schematic demonstrating the measurement of V_L_ for all configurations at 0.5 kΩ. **b** V_L_ of the SA6 configuration. **c** V_L_ of the PA6 configuration. **d** V_L_ of the CC-1 configuration. **e** V_L_ of the CC-2 configuration

In the CC-1 configurations, the combinations [P3 (S2, S2, S2)] and [P2 (S3, S3)] showed a higher V_L_ of 535 mV. In contrast, the combinations [P2 (S4, S2)] and [P2 (S5, S1)] showed a slightly lower V_L_ than the other two combinations (Fig. 8d) but more than that of a single µPSC. In the CC-2 configurations, the combination [S2 (P3, P3)] generated a slightly higher V_L_ than the other combinations (Fig. 8i). However, all the combinations showed a higher V_L_ than the single µPSC.

### Load current (I_L_) at 0.5 kΩ

**Fig. 9:**
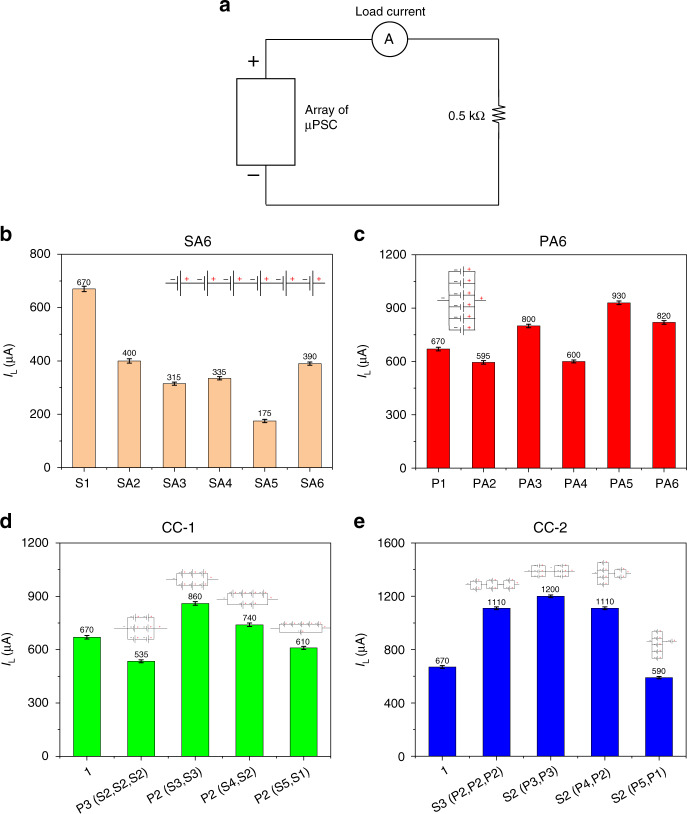
Load current of arrayed micro-photosynthetic power cells at 0.5 kΩ. **a** Schematic demonstrating the measurement of I_L_ for all configurations at 0.5 kΩ. **b** I_L_ of the SA6 configuration. **c** I_L_ of the PA6 configuration. **d** I_L_ of the CC-1 configuration. **e** I_L_ of the CC-2 configuration

Figure 9a shows a schematic of the I_L_ at 0.5 kΩ for all array configurations. In the SA6 configurations, 2 µPSCs to 6 µPSCs in series showed a lower I_L_ than that of a single µPSC. In this configuration, for all combinations, the I_L_ varied from 175 to 400 µA, whereas the single µPSC showed an I_L_ of 670 µA.

In the PA6 configurations, it was found that the I_L_ was slightly higher than that of a single µPSC. In the CC-1 configurations, the combinations [P3 (S2, S2, S2)] and [P2 (S3, S3)] demonstrated a higher I_L_ of 1008 µA and 860 µA, respectively. In contrast, the combinations [P2 (S4, S2)] and [P2 (S5, S1)] demonstrated a slightly lower I_L_ than the combinations [P3 (S2, S2, S2)] and [P2 (S3, S3)] but more than that of a single µPSC. In the CC-2 configurations, the combination [S2 (P3, P3)] generated a higher I_L_ than the other combinations. However, all the combinations showed a higher I_L_ than that of a single µPSC.

### Polarization curve

#### V-I characteristics

The current voltage (I–V) polarization characteristics provide a behavioral understanding of any specific power-generating device.

**Fig. 10:**
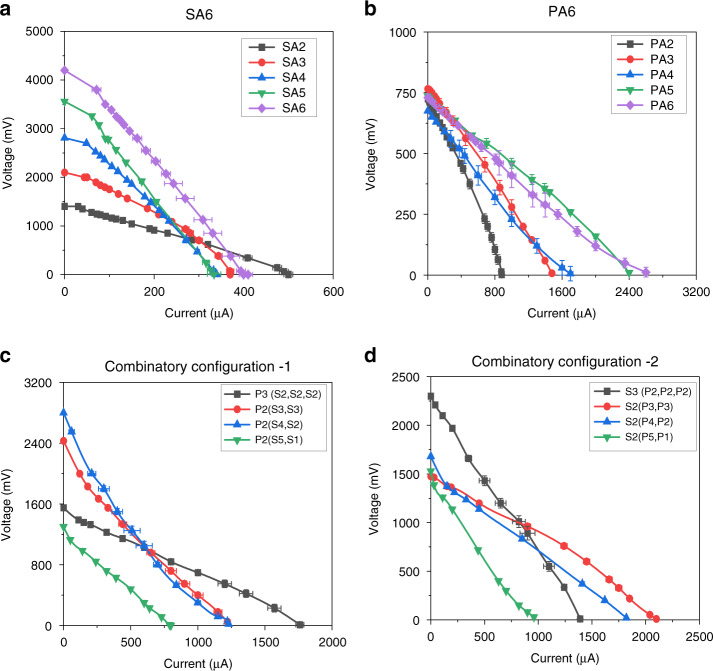
I-V (polarization) characteristics of arrayed micro-photosynthetic power cells. I-V characteristics of all array configurations. **a** I-V polarization curve of the SA6 configuration. **b** I-V polarization curve of the PA6 configuration. **c** I-V polarization curve of the CC-1 configuration. **d** I-V polarization curve of the CC-2 configuration

**Fig. 11:**
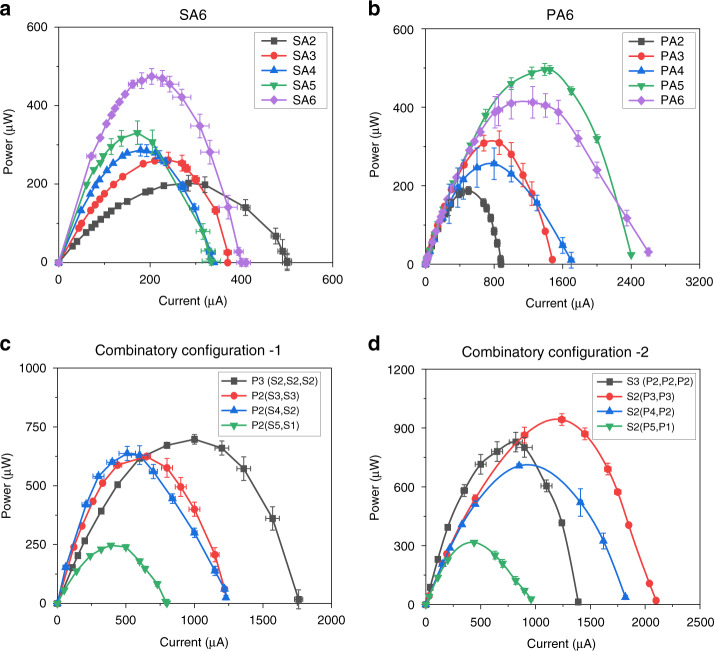
Power-current characteristics of arrayed micro-photosynthetic power cells. Power-current characteristics of all array configurations: (**a**) SA6 configuration, (**b**) PA6 configuration, (**c**) CC-1 configuration, and (**d**) CC-2 configuration

In all the response curves, scales were not uniformly maintained only to display the variation clearly in the plots (Fig. 10). The SA6 configuration area under the curve for the six cells in series was greater than that of two cells connected in series (Fig. 10a). Here, the current remained almost the same, whereas the terminal voltage increased with an increase in the number of µPSCs connected in series. In the PA6 configuration, most of the µPSCs demonstrated almost the same voltage, whereas the current was increased in this configuration. In addition, the area under the curve increased with an increasing number of µPSCs in a parallel configuration. In the CC-1 configuration, the combinations [P3 (S2, S2, S2)] and [P2 (S3, S3)] demonstrated a higher area under the I-V curve than the combinations [P2 (S4, S2)] and [P2 (S5, S1)] (Fig. 11c). In the CC-2 configuration, the combinations [S3 (P2, P2, P2)] and [S2 (P3, P3)] presented a higher area under the I-V curve than the combinations [S2 (P4, P2)] and [S2 (P5, P1)] (Fig. 10d).

#### I-P characteristics

The current–power (I–P) provides the maximum power generated by a typical power source. Figure 11 illustrates the I-P characteristics of the SA6 configurations. As the focus was on array configurations, single µPSC performance was excluded because it was not relevant. However, in our previous works, the I-P characteristics of a single µPSC were presented. In the SA6 configuration, the array of six µPSCs in series showed a higher maximum power (P_mp_) than the other arrays of µPSCs, indicating an increase in the power output with increasing number of µPSCs connected in series (Fig. 11a). However, the current density remained lower than that of the µPSCs alone. In the PA6 configuration, the array of 5 and 6 µPSCs connected in parallel demonstrated higher P_mp_ values. Higher current and higher power were obtained in this configuration because of its higher I_sc_. A P_mp_ of 500 µW and current of 2700 µA were obtained with six µPSCs connected in parallel. Therefore, in the parallel connection, both power and current were increased. However, the voltage of their combinations remained nearly same as the lowest individual µPSC voltage.

**Fig. 12:**
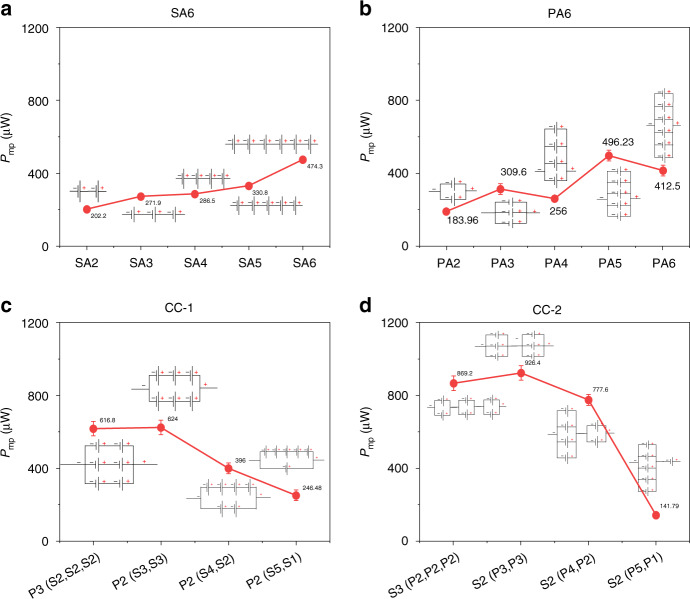
Maximum power of arrayed micro-photosynthetic power cells. **a** Maximum power (P_mp_) of the SA6 configurations. **b** P_mp_ of the PA6 configurations, (**c**) P_mp_ of the CC-1 configurations, and (**d**) P_mp_ of the CC-2 configuration

In the CC-1 configuration, the combinations [P3 (S2, S2, S2)] and [P2 (S3, S3)] demonstrated a higher maximum power (P_mp_) than the other two combinations. This is mainly because of the higher V_oc_ and I_sc_ of these combinations (Fig. 12). In the CC-2 configurations, the combinations [S3 (P2, P2, P2)] and [S2 (P3, P3)] demonstrated a higher P_mp_.

Comparing all four array configurations of CC-1 and CC-2, combinations [P3 (S2, S2, S2)] and [P2 (S3, S3)] showed a higher P_mp_, terminal current and terminal voltage. This result indicated the suitability of utilizing these combinations for real-time low- and ultralow-power applications.

#### Maximum power (P_mp_)

Among the four array configurations, CC-1 and CC-2 demonstrated higher P_mp_ values than the SA6 and PA6 configurations. In the CC-1 configurations, the combination [P2 (S3, S3)] generated a P_mp_ of 624 µW, and [P3 (S2, S2, S2)] produced a P_mp_ of 616.8 µW. In contrast, the other two combinations exhibited slightly lower power. The main reason for the high power of combinations [P2 (S3, S3)] and [P3 (S2, S2, S2)] is the optimal terminal voltage and current of these array configurations. The CC-2 configurations showed higher P_mp_ values than the CC-1 configurations. A P_mp_ of 926.4 µW was generated by the combination [S2 (P3, P3)]. The combinations [S3 (P2, P2, P2)] and [S2 (P4, P2)] generated P_mp_ values of 869.2 µW and 777.6 µW, respectively. A low power of 141.79 µW was generated by the combination [S2 (P5, P1)] because of the poor V_oc_ and I_sc_ of the array combination.

The SA6 configurations showed a P_mp_ of 474.3 µW for six µPSCs connected in series, whereas a P_mp_ of 496.23 µW was generated for five µPSCs connected in parallel. Among the four configurations, the SA6 and PA6 configurations showed lower P_mp_ values than the CC-1 and CC-2 configurations.

The main possible reason for the higher power of the CC-1 and CC-2 configurations is the higher terminal voltage and current of the configuration. In the series connection, voltages were additive, but the current retained the lower µPSC current. Therefore, only voltage increased but not current. In the parallel connection, currents were additive, but voltages remained the same as the lowest µPSC voltage. In the CC-1 and CC-2 configurations with the optimal combinations, both the terminal voltage and currents increased, which resulted in a higher P_mp_. All the data presented in the work are for an electrode surface area of 4.84 cm^2^. By extrapolating these results for a 1 m^2^ area without considering ohmic, concentration, and activation lossesfor the combination [S2 (P3, P3)], the power density is theoretically 1914 mW/m^2^. Similarly, the P_mp_ value of all array configurations for an electrode surface area of 1 m^2^ is shown in Table 2.

**Table 2:** Power density of arrays of µPSCs in mWm^−2^

Array configuration	Maximum power density (mWm^−2^)
Single µPSC	413.2
SA6 (6 µPSCs)	979.9
PA6 (6 µPSCs)	1025.2
CC-1 [P2 (S3,S3)]	1289.2
CC-2 ([S2 (P3,P3)]	1914

The P_mp_ corresponding terminal voltage and current are the operating points of the µPSCs. The operating points are also defined as the ratings of any typical power-generating device. Operating points are essential to design efficient power electronic devices to harness maximum power from the power-generating device. The higher the operating voltage (V_mp_) and current (I_mp_) of the power-generating device are, the better the operating point. With a higher operating point, designing power electronics is relatively cost- and design-effective, and the losses will be lower when real-time loading occurs. Therefore, it is always necessary to have higher operating points for any typical power-generating device. The SA6 configurations demonstrated higher voltages and lower currents; the PA6 configurations showed higher currents and lower voltages; and the CC-1 and CC-2 configurations showed optimal voltages and currents.

From the P_mp_ and operating points (V_mp_ and I_mp_), the CC-1 and CC-2 configurations were found to be better for real-time applications, as their configuration demonstrated both higher terminal voltages and currents.

#### Real-time loading: powering light-emitting diodes

Most low- and ultralow-power devices such as humidity sensors, weather-monitoring sensors, IoT sensors, and many other low-power devices such as biosensors, require work over a short measuring time, interspaced by long periods of latency. For such low-power and ultralow-power applications, µPSCs will be the optimal power source. Thus, to assess the reliability of the µPSC array for one such low-power application, a light-emitting diode (LED) with ratings of 2 V/2 mA and 1.7 V/2 mA was powered for a period of 4 to 6 hours continuously.

Two different LED ratings were tested. In the first case, a 2 V/2 mA green LED was utilized. The first six µPSCs from Table 1 were used to power these LEDs. In the second case, a 1.7 V/2 mA current rating LED was used. µPSCs from 7 to 12 in Table 1 were employed in this case.

**Fig. 13:**
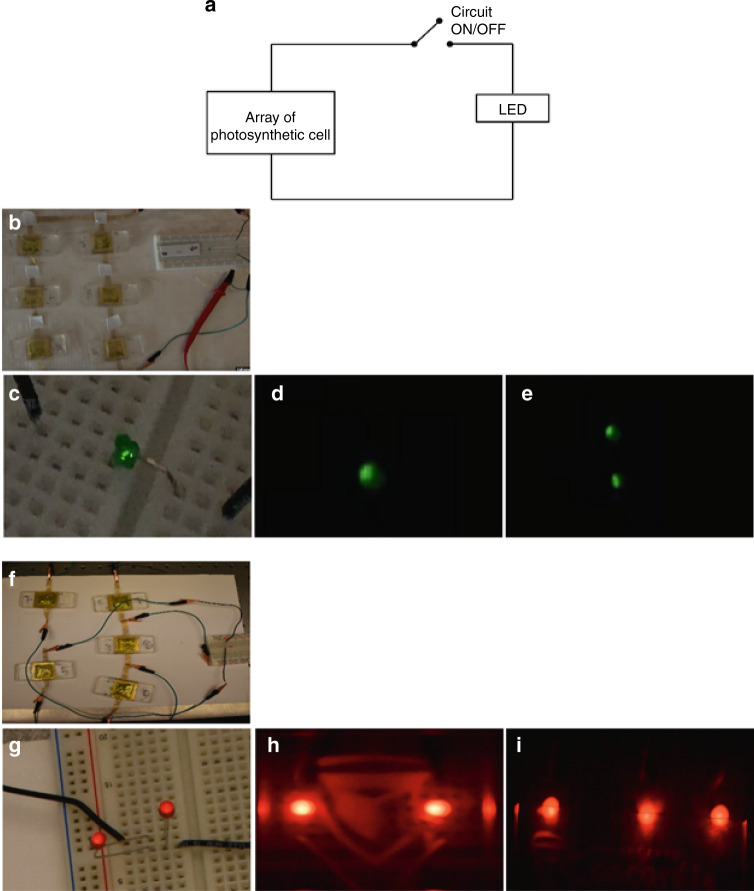
Real time powering of light emitting diodes from arrayed micro-photosynthetic power cells. **a** Schematic of an LED circuit connected to an array of µPSCs. **b** The array combination utilized for the LED-lit, terminal voltage and current of an array of µPSCs, LED rating 2 mA and 2 V. **c** LED lit up in the light condition of 1000 lux; the LED illuminance was low because of the lower current input to the LED. **d** LED glowing from the µPSC in the dark. **e** The two LEDs were connected in parallel in the dark. **f** Photograph of an array combination of 5 µPSCs with a terminal voltage and current of 2.1 V and 1.9 µA, respectively. **g** The two LEDs connected in parallel under the light condition of 1000 lux lit for more than 4 h. **h** The two LEDs glowing from the µPSC in the dark. (i) Three LEDs connected in parallel lit up in the dark

To generate the optimal voltage and current required to power the LED, suitable array combinations were chosen based on performance analysis of all array configurations. The CC-1 configuration’s [P2 (S3, S3)] combination was utilized to powering a 2 V/2 mA LED (Fig. 13) owing to its desirable output voltage and current performance. Alligator clips and single-strand cables served as the connectors. The LED was connected to a breadboard, as shown in the schematic (Fig. 13c, g). After circuit connection, the effective voltage was noted as 2.1 V, and the current was 1.2 mA. Due to losses in the array configurations and ohmic losses due to circuit components, the terminal voltage and the currents were found to be lower. The illuminance of the LED was low because of the lower current from the array of µPSCs. The LED was not powered at full capacity due to the lower supply of current (1.2 mA) from the µPSC than the rating (i.e., 2 mA). Subsequently, two LEDs of the same rating were connected in parallel. Both LEDs were powered by a µPSC array configuration. However, due to the division of the µPSC current between the two LEDs, their luminance was slightly reduced (Fig. 13c). Following this experiment, to observe the response in the dark, the complete dark condition was set up; it was found that for a short duration of light and dark cycles, the performance of the array of µPSCs was unaffected. Moreover, two LEDs were successfully powered in the dark (Fig. 13e). In the second case, only five µPSCs were utilized. First, three µPSCs were connected in series, and second, another set of two µPSCs were connected in series. Finally, both sets were connected in parallel to obtain the desired voltage and current to power up the LED (Fig. 13f). After establishing the array configuration, the effective voltage and current were measured as 1.72 V and 1.8 mA, respectively. For this circuit connection, a lower power rating LED (1.7 V/2 mA) was used. As the power rating of the LEDs was low, an attempt was made to power the two LEDs in an array configuration. Therefore, both similar rating LEDs were connected in parallel.

Both LEDs were successfully powered and emitted moderate luminance (Fig. 13g). Following the light condition response, to test the performance in the dark, the complete dark condition was established. In the short span of light and dark cycles, the performance of array configurations was found to be almost the same. To test the performance in the dark over a considerable period, 30 min was used. In our previous tests, it was found that after 30 min in the dark, the terminal voltage and current of the µPSC decreased slightly. Although the performance in the dark decreases over a long period, the array configuration is still able to power two LEDs with relatively good luminance. As quantifying the luminance was not a focus of the study, it was not measured. Furthermore, to test the performance with a higher loading condition, under the same dark conditions, another LED of the same rating was connected in parallel to the existing two LEDs. All three LEDs were successfully powered by the array configuration with relatively good luminance (Fig. 13i). Finally, to observe the long-term performance, only two LEDs were connected. The two LEDs were successfully powered for up to 4 h continuously without variation in the terminal voltage and current. The process of connection and disconnection of the circuit was repeated several times, demonstrating reproducibility.

### Reliability

The µPSCs from Table 1 (from 7 to 12 µPSCs) were disassembled and cleaned with deionized water after the removal of anolyte and catholyte. Then, the µPSCs were carefully cleaned with soft absorbents on both the anode and cathode sides. A new exponential phase of liquid algal cultures was prepared and used for testing. At that time, LEDs were also successfully powered, and no significant difference in their performance was observed compared to their previous performance, indicating the reliability of the array configuration of µPSCs.

## Discussion

In summary, several array configurations such as series and parallel configurations and combinations of series and parallel configurations were reported in this work. The model photosynthetic microorganism utilized was C. reinhardtii^16,26^ based on its fast growth, its extensive use as a model photosynthetic organism in photosynthetic studies and its excellent exoelectrogenic activity^16,26^. Due to the self-repair capability of this organism, the whole liquid culture of algal cells shows viability for longevity^6,24,27^. Any temporary damage to the cellular components in the cyanobacterial cells will be restored by self-replication functions. Accordingly, this ability to self-replicate the cellular components in cyanobacteria enhances the longevity of algal cells^6,24,27^, thereby increasing the longevity of the microphotosynthetic power cell performance. Therefore, µPSCs containing whole living photosynthetic organisms outperform µPSCs that are based on cellular pigments, with extended stability, better performance, and lower maintenance costs^1,28,29^. In addition to these advantages, designing µPSCs with better flow regimes would enable the photosynthetic microorganisms to undergo self-repair and generate power continuously. Therefore, this technology has the potential for scale-up and could be used in commercial energy production for low-power applications.

A power output of 1914 mWm^−2^ would power most of the commercially available ultralow-power sensors such as humidity sensors, ultrasonic sensors, and global positioning systems (GPSs), whose power requirement ranges from 0.15 mW to 60 mW. In addition, most IoT sensors require power for a short period with a long period of inactivity. Moreover, IoT ultralow-power sensors with continuous operation of the µPSC under both light and dark conditions with suitable power converters will be useful to charge low-power batteries^30,31^.

Currently, we have shown the electrical performance of µPSCs for a few hours of operation. By designing a system for the continuous flow of anolyte and catholyte, the performance could be extended for continuous operation. Another important aspect is that the materials used in fabrication, such as proton exchange membranes (Nafion) and thin metal foils, are environmentally friendly and biodegradable. From a techno-economical perspective, a detailed cost analysis of the technology was performed in our previous work, which demonstrated the economic and environmental advantages of developing µPSC technology. Therefore, with these salient features, after designing µPSCs with a continuous flow of anolytes and catholytes, we can envision future applications of these fuel cells in charging batteries, cellular mobiles, small electronic devices including earphones, e-readers, etc.

## Conclusion

In conclusion, we demonstrated a simple method of fabricating µPSCs and several array strategies for real-time low-power and ultralow-power applications. Due to low power density limitations, to increase the power density, an efficient array strategy was analyzed. The µPSCs were fabricated and connected in four different configurations, series, parallel, series combination, and parallel combination, to enhance the voltage and current of the µPSC. When connected in series, the voltages were found to be the summation of individual terminal voltages, and currents remained that of the µPSC with the lowest value. When connected in parallel, the effective currents were the summation of individual cell currents, and voltage remained that of the µPSC with the lowest value in array combination. In the CC-1 and CC-2 configurations, which are combinations of series and parallel configurations, both the terminal voltage and current were higher than those of the mere series and parallel configurations. By utilizing the [S2 (P3, P3)] configuration, we generated a power density of 1914 mWm^−2^ with 2 mL of anolyte (C. reinhardtii) and 2 mL of [K_3_Fe(CN)_6_]. To demonstrate the configuration for a real-time low-power device, an LED with a rating of 2 V/2 mA was successfully powered for 4 to 6 hours. This novel fabrication method and array strategy will simplify the applications of µPSCs to several low- and ultralow-power applications.

## Materials and methods

### Algal culture

Algal strains (CC-125 wild-type mt + [137c]) were cultured in 50 mL Erlenmeyer flasks in a temperature-controlled system (23 ± 2 °C). The liquid algal cultures were grown in the presence of continuous light illumination in Tri-Acetate Phosphate (TAP)^32^ media. Cells were grown in the presence of constant light illumination of 20 µEm^−2^s^−1^ with white fluorescent tubes (Philips). Erlenmeyer flasks containing algal culture were cultured on an orbital shaker at 90 RPM^33^. The algal culture grown to the exponential phase (48 hours) (Fig. S2) was used for all the experiments for the µPSCs.

### Device operation

The principle of operation of the µPSC is photosynthesis and respiration. Figure S3 shows the principle of operation—more details on the operating principle of the device can be found in the literature^6,18,19^.

### Fabrication

The µPSC device consists of two identical half-cells, each forming the anode and the cathode separated by a proton exchange membrane (PEM). PEM Nafion 117 was treated before performing the fabrication. An aluminum sheet with a thickness of 0.1 mm was purchased from Dexmet Corporation. The 2.4 × 2.4 cm^2^ aluminum metal sheets were sputtered with 40 nm of gold on both surfaces using a sputter coater by Quoram Inc. The adhesiveness of the gold on the surface of aluminum sheets was tested with a tape scrap test.

Nafion was treated based on a previous protocol^34^. Nafion was incubated at room temperature for 12–14 hours to eliminate its moisture content. Furthermore, the metal electrodes were bonded to Nafion by water-resistant adhesive, and a force of 10 kN was applied for approximately 1 hour for the strong bonding of metal electrodes to Nafion. Metal electrodes of size 0.5 × 3 cm were bonded to both sides of the membrane electrode assembly to connect to the external resistor (circuit). The polymer PDMS was utilized for the anode and cathode chambers. PDMS and curing agent at a 10:1 ratio were mixed well and degassed for 10 min to remove all the air bubbles from the PDMS mixture. Then, a brass mold was used for casting to prepare the anode and cathode chambers. The PDMS was poured in the brass mold and incubated at 60 °C for 4 h. Finally, a PDMS mixture at a ratio of 10:1 was used for the final bonding of the membrane electrode assembly and the anode and cathode chambers. A force of 10 kN was applied, and the sample was kept in an oven at 60 °C for 4 h. The cathode chamber was covered with a microscopic cover glass and sealed with hot glue. Figure S4a shows the components of the µPSC and assembly. Figure 2b shows the dimensions of a single µPSC. Figure 2c shows the test setup of the single µPSC. Figure 2a shows a photograph of the assembled µPSC; a zoomed view illustrates the electrode structures.

### µPSC measurement

The terminal voltage of the µPSC was measured using a current sensing unit (DAQ) specially designed to acquire current and voltage from the µPSC^35^. An external variable resistor was connected to the terminals to measure the loading conditions.

The power of the µPSC was obtained by multiplying the terminal voltage and load current of the µPSC.

1 $${{{\mathrm{Power}}}} = {{{\mathrm{V}}}} \times {{{\mathrm{I}}}}$$

### Polarization curve

Polarization characteristics were obtained by recording the terminal voltage under pseudo-steady-state conditions^6^ by varying a variable resistor. The variable resistor was tuned to change the load current from maximum to minimum, and their corresponding terminal voltages were recorded. The load resistance was varied from 0 to 50 kΩ. The internal resistance of the current measuring circuit was taken into consideration in electrical loading. The terminal voltage of µPSC was plotted for the µPSC current (with a density of 4.84 cm^2^ for all the data). For all the measurements, alligator clips and single-strand connecting wires served as connections to the anode and cathode terminals.

### Light lllumination

Artificial light from a white fluorescent bulb of 15 watts (Philips) was maintained at a constant illumination of 20 µEm^−2^s^−1^ at the µPSC.

### Light-emitting diode (LED)

LEDs were purchased from Digikey Inc. The LED millicandela rating was 0.6 mcd, the forward voltage of the LED was 1.7 V, and the current test was 2 mA.

### Terminal connections used and alligator clips

Brass electrodes purchased from Dexmet Inc., and alligator clips purchased from Digikey Inc., served as the connections of the circuits.

## Supplementary information

Supplementary information
