# Supplementary material for: Arraying of microphotosynthetic power cells for enhanced power output
Source: Microsyst Nanoeng. 2022 Mar 14;8:29. doi: 10.1038/s41378-022-00361-7 (PMC8918551; doi:10.1038/s41378-022-00361-7)
Supplement: Supplementary file 1 — Supplementary information [file 41378_2022_361_MOESM1_ESM.pdf]

Arraying of micro photosynthetic power cells for enhanced power output

Kiran Kuruvinashetti[1], Muthukumaran Packirisamy[1*]

Optical-Bio Microsystems Laboratory, Department of Mechanical, Industrial and Aerospace Engineering, Concordia University

Correspondence:

[1*]Muthukumaran Packirisamy, Professor, Mechanical, Industrial and Aerospace Engineering Concordia University, Montreal, Quebec, Canada H3G1M8. Email: pmuthu@alcor.concordia.ca

Supplementary information

Determination of growth of algal culture:

In photosynthetic cells the light absorbing pigments absorb light a specific wavelength. The absorbance at these wavelengths perhaps varies when the amount of pigment changes in the cell. Therefore to understand the growth (optical density) of the photosynthetic cells the wavelength which is out of absorption range of the cells must be selected to comprehend the growth of the photosynthetic cell. Therefore optical density at wavelength 750 nm was chosen to probe the growth dynamics of the algal cells.

At each 12-hour time intervals from 0th hour to 72 hour the absorption intensity of the algal culture was noted down. The plot signifying the growth of the algal culture is shown in the Figure S2.

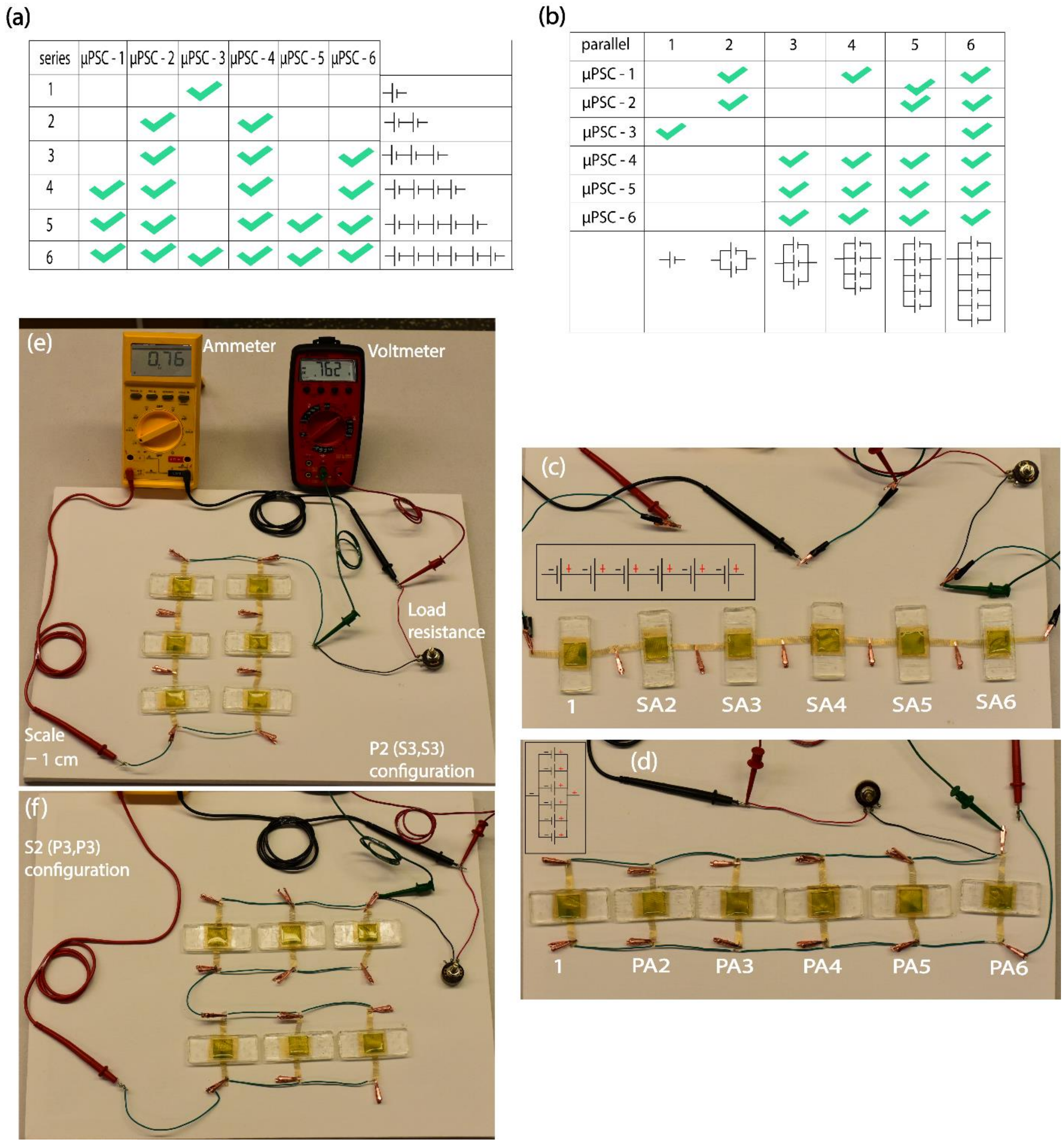

Figure S1: (a) The μPSC utilized in SA6 configuration. (b) The μPSC employed in PA6 configuration of μPSCs (c) The photo image of SA6 configuration (series connection of μPSCs) (d) The photo image of PA6 configurations (parallel configurations of μPSCs) (e) The photograph image showing the [P2 (S3,S3)] configuration demonstrating the measurement of voltage and current. (f) The photograph image of the test circuit connection of [S2 (P3, P3)].

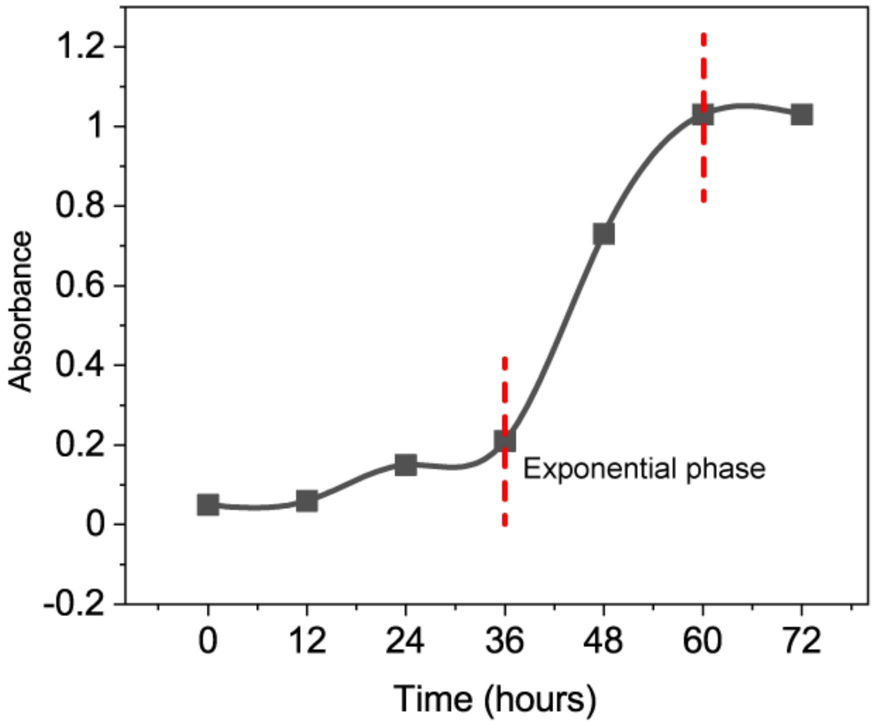

Figure S2. Growth phase of the algal culture from (0th time to 72 hours. From 36 hour to 60 hour the algal culture has attained the exponential phase. Such exponential phase has been utilized for all experiments in the study.)

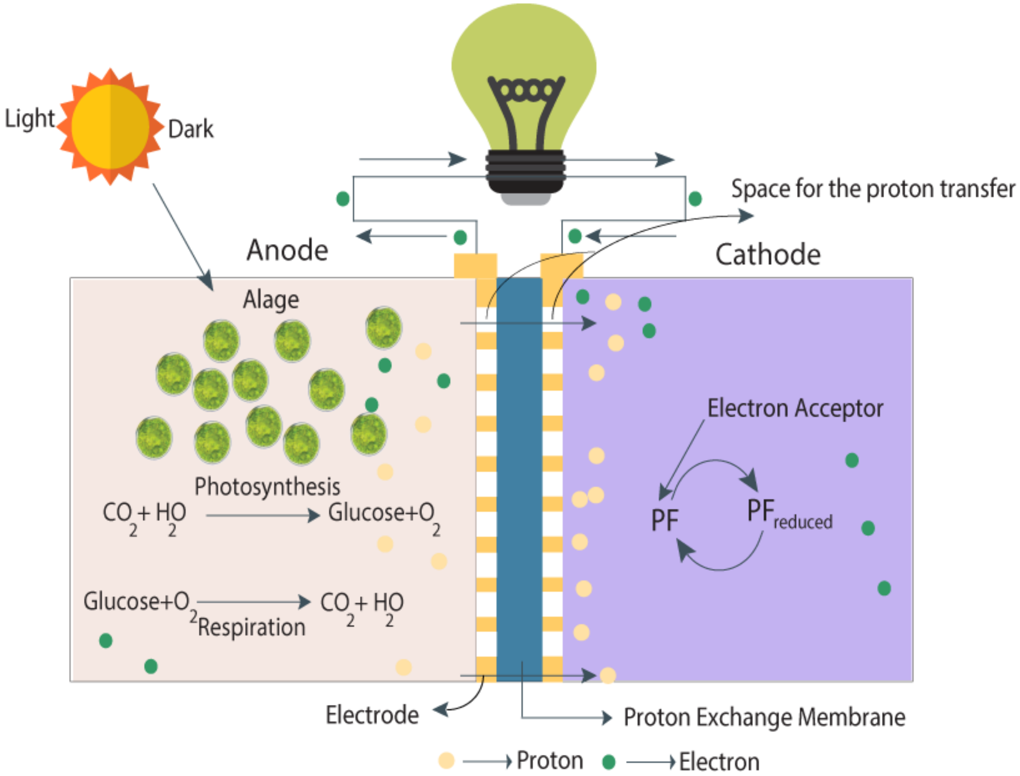

Figure S3: Detailed principle of operation of µPSC

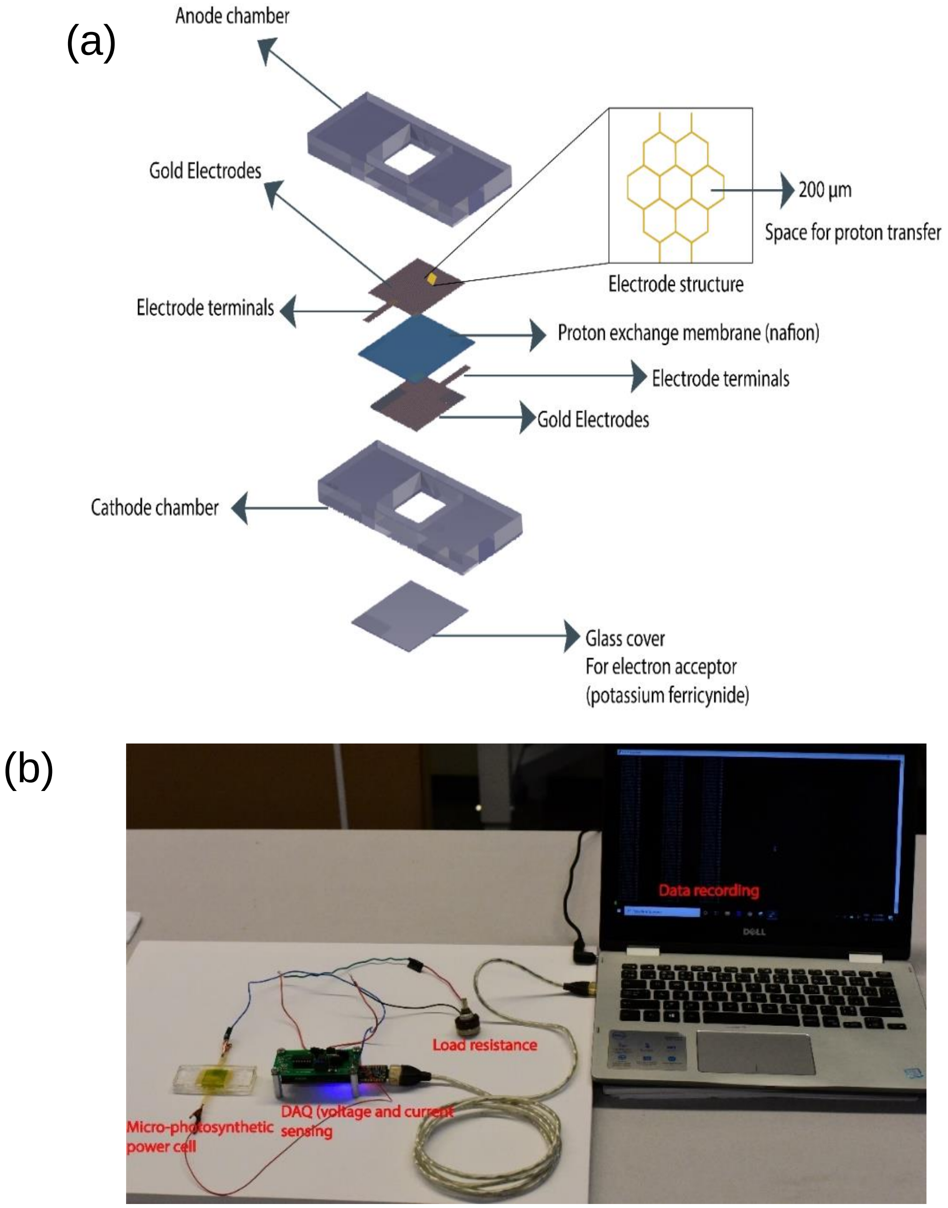

Figure S4: Components of µPSC (b) Testing set up of the µPSC
